# Changes and trends in medication-assisted treatment in Israel

**DOI:** 10.1186/s13584-022-00551-8

**Published:** 2023-01-26

**Authors:** Adi Marom, Iris Levy, Paola Rosca

**Affiliations:** 1 The Hebrew University- Hadassah Medical School, Campus Ein Kerem, 9112102 Jerusalem, Israel; 2 Department for the Treatment of Substance Abuse, Israeli Ministry of Health, 39 Yirmiyahu St., 9446724 Jerusalem, Israel

**Keywords:** Opioid use disorder, Opioid maintenance treatment, Opioid agonist therapy, Medication assisted treatment

## Abstract

**Background:**

As opioid prescription in Israel is increasing, there is a growing need for monitoring opioid use disorder and providing opioid agonist therapy. Our goal is to describe, sub-analyze, and identify obstacles in the treatment of opioid misuse in the Israeli medication assisted treatment centers.

**Methods:**

Data on methadone, buprenorphine, and buprenorphine combined with naloxone for the indication of opioid addiction treatment for the period 2013–2020 were obtained from pharmaceutical companies that distribute them in Israel. Data on utilization of these drugs were also extracted from the database maintained by the Israel Ministry of Health's Pharmaceutical Administration Division. The data were converted to defined daily doses (DDD)/1000 inhabitants/day.

**Results:**

The number of patients receiving medication assisted treatment increased by 10% since 2013, with a shift from buprenorphine alone to buprenorphine/naloxone in government-run centers. Methadone remains the most popular maintenance drug.

**Conclusions:**

The change in opioid maintenance prescription does not match the significant increase in opioid consumption. Optimization of treatment can be achieved by the creation of a comprehensive database, cooperation between healthcare organizations and the government and further development of non-stigmatic and accessible services.

## Background

During the last two decades the use of opioid medications in the US has greatly increased, especially after the introduction of potent opioid pain relievers such as Oxycodone [1]. Physicians' over prescription habits greatly contributed to the opioid epidemic as it was first declared by the Centers for Disease Control and Prevention (CDC) in 2020 [2]. Since then, excessive opioid prescription has also increased in Canada, and at a lesser extent in Europe. Opioid misuse and diversion further expanded to heroin and to illicit synthetic and more potent opioids such as fentanyl and carfentanyl [1]. More than 42,000 Americans died of opioid overdose in 2016 and in 2017 the number of fatalities reached 70,000 [3, 4].

The largest opioid prescription increase among the Organization for Economic Co-operation and Development (OECD) countries in the period 2011–2016 occurred in Israel with a 125% increase [5]. Opioid analgesics are relatively available in Israel: as a part of the government- subsidized Israeli Health Basket, any physician and pharmacy can prescribe and dispense them. This policy is in accordance with the United Nations Office on Drugs and Crime (UNODC) General Assembly on Drugs Policy in 2016, which recommended reducing barriers to pain medications while balancing the risk of overdose, misuse and addiction [6]. Unlike most health services in Israel which are provided by health maintenance organizations (HMO), the treatment of substance use disorders remains under the responsibility of the Department for the Treatment of Substance Abuse in the Ministry of Health.

### Prescription of opioid analgesics in Israel

Opioid prescription in Israel has increased by 47% between 2000–2008, [7] and by 68% between 2009–2016, mainly due to a rise in the consumption of oxycodone and fentanyl [8]. Similarly, data published in 2009 [9] reported a sharp increase in the purchase of opioid analgesics and a 60% increase in the number of patients using opioid medications from Clalit, the largest HMO in Israel. Interestingly, at that time these data were interpreted as positive ones, indicating that people suffering from severe pain would have easier access to treatment by opioid analgesics. Data from Israel revealed a decline in mortality with much lower rates of opioid-related deaths than those reported in the United Kingdom and the United States [10, 11]. Most opioid analgesics are prescribed by family physicians, sometimes after initiation of therapy by hospital or specialist doctors, such as oncologists and orthopedic surgeons. A shortage of pain specialists in the HMOs may contribute to suboptimal pain management and over-prescription of opioids.

### Treatment policy of substance use disorders

In accordance with the Israeli National Health Insurance law [12], all citizens have free medical insurance in one of the four HMOs, which provide most health services. However, the treatment of substance use disorders remains under the responsibility of the Department for the Treatment of Substance Abuse in the Ministry of Health. Recently the Knesset deliberated that these services will be delegated to the HMOs in the future. Harm reduction treatment options first became available in Israel in the 1970s with the introduction of methadone maintenance therapy [13], and have since expanded to treatment centers which offer combined medical and psycho-social interventions. These centers are co- supervised by the Ministry of Health (MOH), and the Ministry of Labor, Social Affairs and Social Services. There are also private office-based medical clinics which are routinely supervised and required to meet the standards of the Department for the Treatment of Substance Abuse [14].

There are 13 public residential detoxification centers for substance use disorder, which combine medical therapy and social support. Each of these contain 4–25 beds and accept up to 550 patients per year. On average, 55% of the patients complete the 3-week detoxification (sometimes up to 3 months). Men and women are treated separately, and some centers specialize in specific population groups, such as dual diagnosis patients, transgenders, Arabs, and orthodox Jews. The centers aim for complete detoxification; but consistently with the harm reduction approach, and in the view that opioid dependence is a chronic disease, some patients are referred to long term medication assisted treatment (MAT) centers for a long-term treatment, which can be lifelong. This enables patients to maintain a good quality of life and daily functioning, and sometimes even achieve complete rehabilitation. The long-term treatment is offered by thirteen ambulatory MAT centers, which deliver a holistic treatment approach, combining social and medical support, including prevention and treatment of infectious diseases [14]. There is long standing evidence that MAT reduces morbidity and mortality [15], and specifically methadone maintenance treatment has been shown to improve survival in an Israeli MAT center [16].

### Long term opioid agonists

The long-term opioid maintenance drugs are only authorized in Israel in combination with psychological and social support. The patients are evaluated by a multidisciplinary team, which includes physicians with experience in addiction, pharmacists, nurses, social workers, psychotherapists, and counsellors. The patients take part in individual and group therapy in the spirit of Motivational Enhancement, cognitive behavioral therapy, trauma focused relapse-prevention and the twelve-steps program. They also receive dental care, are treated for other co-existing addictions, and encouraged to take part in vocational and professional rehabilitation programs. The patients' families also receive counselling.

There are several authorized opioid maintenance drugs in Israel. Methadone, which has been in use since 1970, has proved efficacy in the reduction of the use of illicit drugs and infectious diseases, and is considered the best maintenance drug in terms of patient adherence [17]. However, its drawbacks are risk of overdose, abuse (including third party trade), and long QT arrhythmias, although QTc prolongation is uncommon and rarely leads to cardiac pathology [18, 19]. In Israel methadone is only used in government run MAT centers [14].

Buprenorphine is a preferable treatment option because of a reduced risk of overdose, and a long half-life. In addition, it is available in several forms of administration and fixed doses, as opposed to methadone which requires pharmacy preparation. Buprenorphine is prescribed and supplied only by certified doctors and pharmacies. Buprenorphine/naloxone fixed- dose combinations are available as sublingual tablets (Suboxone®) and sublingual film (Suboxone Film®). The combination of naloxone, a full opiate antagonist, decreases the risk of abuse since it is only activated in off label administration routes. The sublingual tablets, which are available since 2013, are free of charge in public clinics. The sublingual film was introduced in 2019 as a pilot for 105 patients. Due to its rapid onset, improved taste, and a reduced risk of abuse, patients in public clinics were planned to gradually switch from tablets to film in 2020 [14]. As of October 2021, 95% of patients are currently on film.

In 2019 Israel was the fourth country to register extended-release buprenorphine for a monthly abdominal subcutaneous injection (Sublocade®). This formulation is expected to reduce illegal trade and misuse, as well as minimize stable users' contact with rehabilitation centers and promote their autonomy and rehabilitation. The drug is available to patients who are stabilized on a trans-mucosal buprenorphine-containing product. Newer patients attend the clinic at least twice a week, while stable patients arrive monthly to receive Sublocade® or four weekly prescriptions of buprenorphine.

This article aims to describe how Israel copes with opioid use disorders, to sub-analyze data on consumption patterns of opioid maintenance drugs throughout 2013–2020, and to identify obstacles in diagnosis and optimal delivery of treatment. We will also describe recently opened innovative and non-stigmatic services for chronic pain patients who became addicted to opioid analgesics.

## Methods

### Data collection

Data on the total annual administration of the opioid maintenance medications (methadone, buprenorphine, buprenorphine/naloxone, both tablets and film) for the years 2013–2020. were derived from the database maintained by Sarel: the largest private supplier of drugs to the Israeli Ministry of Health and the only provider of opioid maintenance drugs to public institutions. We also extracted data from the database maintained by SLE, a logistic center for pharmaceuticals and medical equipment which is the only provider of opioid maintenance drugs to both Sarel and private pharmacies. In turn, those pharmacies supply the drugs to private institutions.

Data on the total population of opioid maintenance patients for each year were obtained from the database maintained by the Department for the Treatment of Substance Abuse in the Ministry of Health, which only contains information on public clinics and residential detoxification centers.

This retrospective study did not require Institutional Review Board approval because the data are anonymous and do not contain any identifiable codes. The data on the Israeli population of patients is available from the Department for the Treatment of Substance Abuse in the Ministry of Health.

### Sub-analysis

Dosages for all drugs were converted to defined daily doses (DDD) per 1000 inpatients per day, which is the average maintenance dosage as defined by the World Health Organization Collaborating Center for Drug Statistics. DDDs are based on the ATC classification index [20].

To calculate administration rates, we used the formula "number of DDD per 1000 opioid maintenance patients per day = number of packages dispensed × number of doses per package × number of mg per dose × 1000 patients/DDD in mg × number of opioid maintenance patients in Israel for the year × 365 days".

## Results

Over the study period, the number of patients receiving MAT in Israel’s national health care system increased by 11%, from 3444 in 2013 to 3864 in 2020 (Table 1). In the study period there was a shift from buprenorphine alone to buprenorphine/naloxone. In 2013 only 1.95% of buprenorphine users in public facilities received naloxone, in contrast to 93.94% in 2019. This shift occurred between 2014 to 2015: in 2014, 10% of buprenorphine users received naloxone, and in 2015 the percentage rose to 94%. Throughout the years, methadone remained the most popular maintenance drug, prescribed to 84–87% of the patients in the public system.

**Table 1:** Number of public health care patients by opiate maintenance drug

	2013	2014	2015	2016	2017	2018	2019	2020
Buprenorphine: total	461	618	570	480	633	679	693	758
Buprenorphine alone	452	556	34	5	38	41	42	38
Buprenorphine/Naloxone	9	62	536	475	595	638	651	720
Methadone	2983	3623	3474	3344	3344	3209	3109	3106
Total	3444	4241	4044	3824	3977	3888	3802	3864

**Fig. 1:**
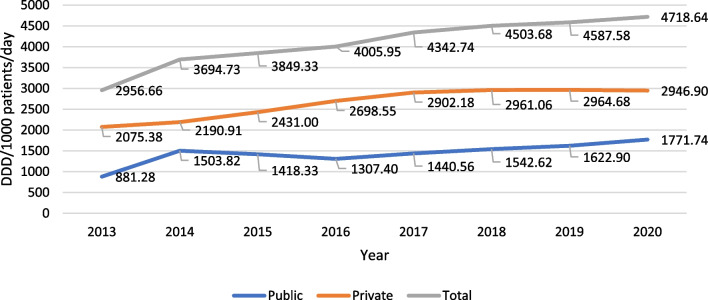
Buprenorphine and buprenorphine/naloxone use 2013–2020 in private and public centers

Total consumption of Buprenorphine products increased by 156%, from 2956.66 to 4718.64 DDD/ 1000 patients/day (Fig. 1). Consumption in private office-based clinics rose by 142%, from 2075.38 to 2946.90 DDD/ 1000 patients/day. Consumption in public clinics increased by 201%, from 881.28 to 1771.74 DDD/ 1000 patients/day. In average, private office-based clinics used about twice the amount of Buprenorphine relative to public ones (average public: private yearly ratio 0.54).

**Fig. 2:**
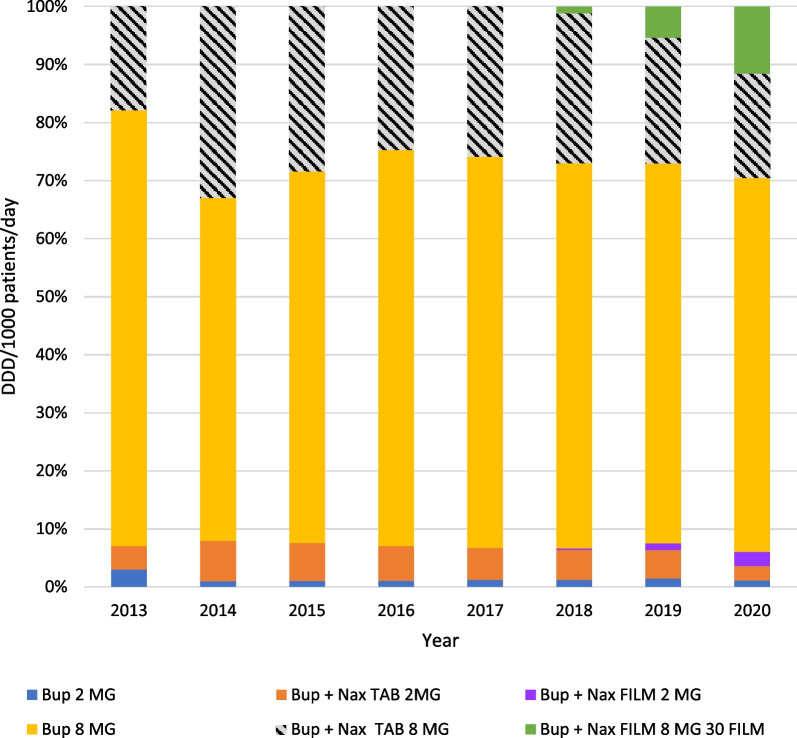
Relative consumption of buprenorphine products 2013–2020

Analysis of the consumption trends across different types of Buprenorphine products (Fig. 2) shows that Buprenorphine 8 mg is the most used drug, although its relative part decreased from 75.05% on 2013 to 64.38% in 2020. Most physicians in Israel prescribe a daily maintenance dose of 16 mg of Buprenorphine, as is advised in various guidelines [21, 22]; the data, however, was analyzed by the World Health Organization's DDD of 8 mg.

In accordance with the MOH's plans, the relative consumption of buprenorphine/naloxone film increased from 6.52% of all buprenorphine consumption to 13.98% in 2020.

## Discussion

In this paper, we aimed to sub-analyze how MAT in Israel has changed and evolved during the last years, and to identify obstacles to an optimal treatment. Our data show that the number of patients treated for opioid use disorder in public facilities increased by 11% over the study period, which is proportional to the growth in Israel's population. There is no Israeli database collecting information on the prevalence of opioid medication use disorder in medical centers, including HMOs; however, according to Ponizovsky et al. [8], from 2009 to 2016 there was a fourfold increase in the consumption of oxycodone, and a twofold increase in the consumption of fentanyl. They explain these findings by an increase in the number of patients using prescribed opioids and the average duration of consumption per patient. Therefore, an increase in opioid misuse and dependence may be underdiagnosed, undetected and untreated.

We have identified two main challenges in the treatment of opioid use disorders in Israel: accessibility to treatment, including considerations of special populations, and a lack of a reliable and consistent national database assembling aggregated data on opioid usage both from the HMOs, hospitals, and MAT centers. This impairs both identification of potential patients and access to data essential for supervision and policy updates.

Addiction medicine is not an inherent part of the general Israeli health system. Originally, the National Health Insurance Law did not include any mental health services. This was amended in 2015 with the Psychiatric Reform, when the operation of these services was delegated to the HMOs, but the treatment of substance use disorders remained until now under both the supervision and the subsidization of the Ministry of Health. This causes fragmentation of treatment and impairs proper continuity of care. Access to addiction specialists requires referral from the HMOs to the government-run facilities. These two bodies maintain different and unconnected databases. We hope that with the recent Knesset decision, this situation will improve in the near future.

Until 2017, the MAT centers treated mostly patients belonging to criminal, homeless, and other stigmatic populations. Chronic pain patients tend to develop an iatrogenic addiction, as their use of opioid analgesics is generally initiated after traumas, accidents, or orthopedic diseases. Therefore, the stigma associated with MAT centers constitutes a real barrier to treatment. The Department for the Treatment of Substance Abuse endeavors to reduce the stigma associated with their services; for example, in 2019 two MAT centers moved to renovated buildings which are centrally located and easily accessible by public transportation. In addition, separate and dedicated treatment pathways were initiated in MAT facilities for the population of persons addicted to prescription opioids.

Since 2017, two HMOs (Maccabi and Meuhedet) have opened their own clinics for the treatment of prescription opioid medication use disorder. As a part of the regular services available to HMO members, these clinics are less stigmatized and more approachable. The HMO clinics offer assessment and diagnostic services, and a gradual detoxification for patients who do not suffer from chronic pain. Chronic pain patients are gradually stabilized on buprenorphine, and after a year referred to a government-run MAT center. Chronic pain patients can approach the MAT centers in separate hours from those who use illicit drugs. In 2019 there was a rise in the admittance to MAT centers, which may be attributed to this population of patients [14].

It is our belief that the HMOs should be encouraged to take a larger part in the diagnosis and treatment of prescription drugs misuse. In addition to geographically and conceptually accessible clinics, the HMOs possess data on individual prescription and consumption patterns, medical background, and comorbidities. Since the HMOs and the MAT centers use separate databases, each institution receives only patient-reported information about the other, which may impair continuity of care and lead to suboptimal medical and psychiatric-psychosocial care. For example, a patient may paradoxically receive both opioid analgesics from the HMO and buprenorphine from the MAT center.

Another instance which requires communication between MAT centers and general hospitals is continuity of administration of opioid maintenance treatment in hospitalized patients. The Department for the Treatment of Substance Abuse encourages general hospitals to continue buprenorphine treatment; during 2019 it was prescribed to patients in nine general hospitals and five psychiatric ones [14]. Efforts should be made to expand the treatment to more hospitals. Under-prescription of MAT to hospitalized patients is also prevalent in the US [15], where the stigma against people with substance use disorders and strict regulations on the prescription of buprenorphine have been suggested as major factors.

Despite its drawbacks, the maintenance of separate databases increases patient confidentiality and may encourage patients to seek treatment without the risk of stigma from other healthcare providers. The Ministry of Health's operation of substance use disorder services also guarantees the allocation of funds and multidisciplinary healthcare teams to this specific cause. The MOH provides professional training for caregivers: a 2-day course for physicians from various disciplines on buprenorphine treatment principles, specific courses on Sublocade® and Suboxone Film®, and workshops on motivational interviewing, de-escalation for coping with violent patients, dual diagnoses, and suicide prevention.

The data on people who receive MAT does not differentiate between illicit drug users to people who have become dependent on prescription drugs. A Parliament report from 2017 found that the four HMOs differ in their definitions of prescription drugs dependence and misuse, and all these organizations acknowledge that their data is partial. Three of the HMOs defined an addiction by a combination of an International Classification of Diseases (ICD) 9 diagnosis and drug purchase over differing periods of time. The differing definitions make comparison difficult; there is a pressing need for a nationwide documentation and monitoring system for opioid prescription and distribution. In 2019, the General Manager of the Ministry of Health allocated a specialists' committee to tackle this growing problem and determine on a uniform way of half-yearly data transfer from the HMOs to the Ministry of Health on problematic opioid medication use. The committee's recommendations are still pending.

It has been noted that although utilization of potent opioids in Israel is increasing, opioid-related morbidity and mortality are decreasing [11]. Possible explanations are the HMOs' oversight on prescribers, compliance of pharmacists with dispensing limits and the subsidization and availability of MAT. As opioid-overdose reported deaths have declined steadily from 56 in 2009 to 17 in 2017 [14], a different and worrying explanation is that adverse events are under- reported, under- diagnosed or reported as sudden cardiac death. This may be partly due to the complexity of the ICD's classification methodology [23], but also to the deficient documentation on substance abuse. Data on 2020–2021 opioid- related deaths in Israel is not yet available, but data from around the world shows an increase during the Covid-19 pandemic [24, 25]. It remains to be verified whether the same applies to Israel also.

Another accessibility problem is to buprenorphine itself: in contrast to opioid analgesics, it can only be prescribed for addiction treatment by physicians accredited by the Department for the Treatment of Substance Abuse and supplied by 22 certified pharmacies. Each patient is enrolled at a specific pharmacy and cannot receive their medication elsewhere. Usually, chronic medications in Israel are dispensed for 3 months ahead to ameliorate the burden of disease. Buprenorphine was prescribed weekly to prevent abuse and diversion until March 2020, when the Covid-19 pandemic caused the MOH to allow monthly prescriptions until June 2021. The MOH now considers allowing monthly prescriptions terminally to improve adherence to prolonged treatment. Interestingly, with a similar agenda in mind, in April 2021 the US has eased the certification requirements for healthcare practitioners to buprenorphine prescription [26].

Our results show that between 2014 and 2015 there was a shift from buprenorphine alone to buprenorphine/naloxone. The growing prevalence of naloxone should encourage the MOH to allow long term prescriptions. The Pharmacists' Regulations on Dispensing and Transferring Drugs (1983) define a small number of drugs which cannot be supplied for more than a week. Buprenorphine is not included in these regulations, which means that there is no legal basis to the current prescription limitations. As most Israeli patients receive naloxone-containing products, the risk of diversion is relatively small, and measures to improve access to buprenorphine should be considered.

Access to methadone may also be challenging, as it is only available in Israel in government-run clinics, while in some other countries physicians may choose freely based on individual medical and psychosocial factors. For instance, Canadian guidelines state that in some conditions, such as patient preference and a challenging induction, methadone may be a more appropriate choice [27]. Our results show that methadone remains the most widely used MAT drug in Israel. A possible explanation for this may be that as a full opioid agonist, methadone is more effective than at treating withdrawal symptoms than buprenorphine, which is a partial agonist. Discontinuing methadone causes a withdrawal syndrome that may discourage patients from switching to buprenorphine [17]. Also, methadone may be preferred because of its analgesic and euphoric effects [28].

It should be emphasized that despite the possible hassle of getting to a specific pharmacy, opioid agonists are at least financially accessible in Israel, as they are subsidized by the government. This is a strength of the Israeli health system, as the high cost of medication is a real barrier in the treatment of opioid use disorder; an Australian study, for example, found that participants spent one eighth of their income on maintenance therapy [29].

The other aspect of the accessibility issue is to opioid analgesics, which are, perhaps paradoxically, easier to obtain than buprenorphine. As most opioid analgesics are prescribed by family physicians, they should possess knowledge about pain medication regimes and how to choose wisely between the different options. The Israeli Medical Association also emphasizes the need to evaluate patient and disease characteristics before prescribing opioid analgesics, and also describes warning signs for misuse [30].

In 2019, the Israeli Ministry of Labor, Social Affairs and Social Services estimated that 120,000 Israelis suffer from substance use disorders, only 20% of whom are treated. A State Comptroller report from 2019 noted that a main deficiency in substance use disorders treatment is a lack of continuity between emergency departments, community medicine and social services. In accordance with this study's results, the Comptroller implies that the public health system fails to meet the needs of people with substance use disorders, in terms of clinics availability, accessibility and reaching out to potential patients [31].

There are several tools for the early detection of opioid misuse, for example the self-reported Current Opioid Misuse Measure, designated for chronic pain patients [32], and a machine learning classifier intended for emergency departments [33]. The latter weighs patient characteristics such as chronic pain and a history of trauma, intoxication, and substance use. A similar tool which also considers the dose and duration of opioid treatment may aid family and community physicians in detecting high risk patients.

This study has a few limitations. Since there is no reliable data on actual drug consumption, we used logistic periodic data on drug sales. This data may differ from real consumption habits, for example in cases of non-adherence and expired drugs. Also, our sub-analysis was preformed from the point of view of policy makers; we did not interview healthcare providers working in MAT centers on the challenges they face, and the drawbacks and strengths of the current policy.

## Conclusions

There is a discrepancy between the significant increase in the prescription of opioid analgesics and the minor rise in the number of MAT patients in Israel. The Department for the Treatment of Substance Abuse supervises residential detoxification clinics and MAT centers, and in cooperation with the HMOs endeavors to address the needs of patients who are addicted to prescription opioids and to develop non-stigmatic services. Treatment of opioid use disorder in Israel can be optimized by a uniform and organized database; improving access to MAT including long term prescriptions; and the integration of the treatment of substance use disorders into the National Health Insurance services, which will allow earlier diagnosis, continuity of care and data analysis.

## Data Availability

Raw data were generated from Sarel and SLE drug wholesalers; the datasets are available from the corresponding author on reasonable request.

## Abbreviations

DDD: Defined daily dose
CDC: Centers for Disease Control and Prevention
OECD: Organization for Economic Co-operation and Development
UNODC: United Nations Office on Drugs and Crime
HMO: Health Maintenance Organization
MOH: Ministry of Health
MAT: Medication assisted treatment
